# Efficient BFCN for Automatic Retinal Vessel Segmentation

**DOI:** 10.1155/2020/6439407

**Published:** 2020-09-17

**Authors:** Yun Jiang, Falin Wang, Jing Gao, Wenhuan Liu

**Affiliations:** College of Computer Science and Engineering, Northwest Normal University, Lanzhou, Gansu, China

## Abstract

Retinal vessel segmentation has high value for the research on the diagnosis of diabetic retinopathy, hypertension, and cardiovascular and cerebrovascular diseases. Most methods based on deep convolutional neural networks (DCNN) do not have large receptive fields or rich spatial information and cannot capture global context information of the larger areas. Therefore, it is difficult to identify the lesion area, and the segmentation efficiency is poor. This paper presents a butterfly fully convolutional neural network (BFCN). First, in view of the low contrast between blood vessels and the background in retinal blood vessel images, this paper uses automatic color enhancement (ACE) technology to increase the contrast between blood vessels and the background. Second, using the multiscale information extraction (MSIE) module in the backbone network can capture the global contextual information in a larger area to reduce the loss of feature information. At the same time, using the transfer layer (T_Layer) can not only alleviate gradient vanishing problem and repair the information loss in the downsampling process but also obtain rich spatial information. Finally, for the first time in the paper, the segmentation image is postprocessed, and the Laplacian sharpening method is used to improve the accuracy of vessel segmentation. The method mentioned in this paper has been verified by the DRIVE, STARE, and CHASE datasets, with the accuracy of 0.9627, 0.9735, and 0.9688, respectively.

## 1. Introduction

Ophthalmology is an important research area of contemporary medicine. Eye health is closely related to people's lives. There is a wide variety of ophthalmic diseases, such as cataract, glaucoma, and diabetic retinopathy that have a high incidence, and diabetic retinopathy is one of the main causes of blindness [1]. Because retinal blood vessels provide the only noninvasive view of the cardiovascular system, they are the key feature that can be referenced for the diagnosis of ophthalmic diseases [2]. The main structure of a normal retinal fundus image is the optic disc, macular, and blood vessels. Hard exudation, soft exudation, microaneurysm, and other structures may be observed in the fundus image of the diseased retina. The morphology of blood vessels is a key indicator for early detection of retinal disease and understanding of the severity of the disease. Ophthalmologists usually perform blood vessel segmentation manually through retinal images to extract lesion information. However, even for an experienced doctor, this work is cumbersome, error-prone, and time-consuming [3].

In recent years, with the development of computer vision technology, many fundus blood vessel analysis methods have been proposed [4–21]. The computer can quickly, automatically, and accurately segment retinal blood vessels, which will greatly improve the diagnosis rate and work efficiency of doctors. There are two major types of the analysis methods: supervised learning method and unsupervised learning method [22]. The unsupervised learning method is designed based on the inherent properties of blood vessels and does not need to refer to manually labeled tags. However, compared with the supervised learning method, there are some problems with the unsupervised learning method. Due to noise and pathological patterns, the performance and generality of the unsupervised method are poor. Morphological processing can segment the vascular structure, but it must be combined with other methods to obtain accurate results [4]. Hoover et al. proposed a threshold detection technique of a matched filter response image. This method can complement the local blood vessel attributes with the region-based network attributes to achieve the purpose of segmenting blood vessels [5]. Several studies used matched Franci and Gabor wavelet filters, both individually and together, to enhance blood vessels and improve segmentation [6–8]. Saffarzadeh et al. used multiscale methods to segment blood vessels, but small blood vessels with low contrast cannot be detected [9, 10]. Roychowdhury et al. [11] proposed a region growing method for segmenting blood vessels, but specialized knowledge was required in the setting of blood vessel seed points and the formulation of termination rules. By combining a trainable B-COSFIRE filter with an adaptive threshold method, Ali et al. [12] proposed the improvement over the current method of retinal blood vessel segmentation. The proposed method can automatically configure selectivity in a prototype mode check. Chen [13] proposed a novel hybrid active contour model for automatic segmentation of fundus images.

The supervised learning method for retinal blood vessel segmentation using label data includes two steps: (1) blood vessel feature extraction and (2) pixel classification. Wang [14] proposed a method for segmenting retinal blood vessels in color fundus images based on supervised learning, using a nonlinear support vector machine (SVM) classifier to classify image pixels into vascular and nonvascular. *K*-nearest neighbor (*K*NN) classifier is used for soft segmentation of retinal blood vessels, classifying each image pixel as blood vessel or nonvascular to generate the final segmented image [15]. Compared with the traditional neural network, U-Net [16] with the fully convolutional neural network (FCN) structure has attracted more attention due to its ability to obtain from coarse to fine representation. Fu [17] proposed a method of the convolutional neural network (CNN) combined with the fully connected conditional random field (CRF) to perform retinal blood vessel segmentation. Li [18] proposed a wide and deep neural network with strong induction ability to segment retinal images. Liskowski and Krawiec trained CNNs with fundus image patches, which were preprocessed by zero-phase whitening, global contrast normalization, and gamma correction [19]. Lin et al. [20] proposed a deep learning method combining global nested edge detection and the conditional random field. In the study by Samuel and Veeramalai [21], a multilayer/multiscale deep supervised layer technique was proposed to better segment retinal blood vessels.

Low-quality and artefact-ridden images can affect the performance of segmentation methods. Therefore, the proposed models usually have the following problems [23]: (1) the downsampling factor of the model is too large, which leads to the feature information of a large number of small blood vessels that is lost in the retinal image, and the information eventually cannot be recovered; (2) the receptive field of the model is too small, which leads to insufficient understanding of local context information, and it is impossible to accurately distinguish pathological regions and blood vessels in the retinal image, causing the incorrect segmentation; (3) the feature extraction capacity of the network structure is insufficient, it is difficult to restore low-level detailed feature information, and a lot of noise is generated in the segmented blood vessel image; and (4) the inability to obtain the accurate information of blood vessels of different sizes results in the inability to accurately detect blood vessel edges and small blood vessels.

In view of the above issues, this paper proposes a retinal blood vessel segmentation model based on the deep FCN. The main work is as follows:

1. An image preprocessing method based on automatic color enhancement (ACE) technology is proposed to improve the image quality, make the vascular area more obvious, and achieve better segmentation results.
2. An improved deep FCN called the butterfly full convolutional neural network (BFCN) for automatic segmentation of retinal blood vessels. Compared with basic the FCN, the BFCN has the following advantages: (i) multiscale input can effectively improve the quality of segmentation; (ii) using dilated convolution with different expansion rates to obtain larger receptive fields and rich spatial information is helpful in fully understanding local context information; and (iii) the transfer layer performs the global average pool on the output of the encoding path and calculates the attention vector to guide the feature map learning. It can improve the network's sensitivity to information features. In the absence of any supervisory information, the feature information is of great significance to the decoder, and effective encoder information can make better predictions.
3. A sharpening method is brought forth to postprocess the predicted segmented image to improve the accuracy of retinal vessel segmentation.

The paper proceeds as follows.  Section 2 of the paper mainly expounds the aforementioned method.  Section 3 outlines the results and validates the proposed BFCN model.  Section 4 summarizes the proposed methods.

## 2. Method

### 2.1. Datasets

**Figure 1:**
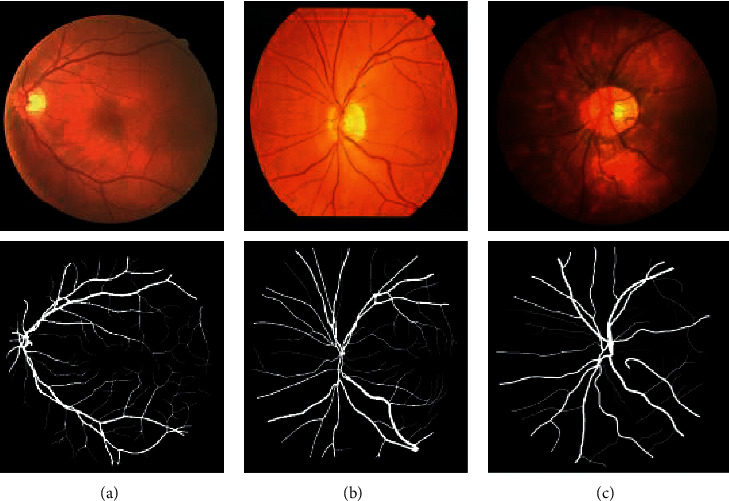
The dataset used. The first row is the original image, and the second row is the ground truth. (a) DRIVE, (b) STARE, and (c) CHASE.

This paper used three public datasets: Digital Retinal Images for Vessel Extraction (DRIVE) [24], Structured Analysis of the Retina (STARE) [5], and CHASE_DB1 Retinal Image Database (CHASE) [25] for blood vessel extraction and to verify the performance of the BFCN model.  Figure 1 shows an original picture and corresponding ground truth in these three datasets.

The DRIVE dataset consists of 40 retinal fundus blood vessel images, corresponding ground truth images, and corresponding masks images from the diabetic retinopathy screening program in the Netherlands. The size of each image is 565 × 584 (http://www.isi.uu.nl/Research/Databases/DRIVE/).

The STARE dataset consists of 20 retinal fundus blood vessel images, corresponding real labeled images, and corresponding masks images. Each image is digitized to 700 × 605 pixels (http://www.ces.clemson.edu/ahoover/stare/).

The CHASE dataset consists of left and right eye fundus images, corresponding real labeled images, and corresponding masks images of 14 students. There are 28 images in total, each with a resolution of 1280 × 960 (https://blogs.kingston.ac.uk/retinal/chasedb1/).

### 2.2. Image Preprocessing

**Figure 2:**
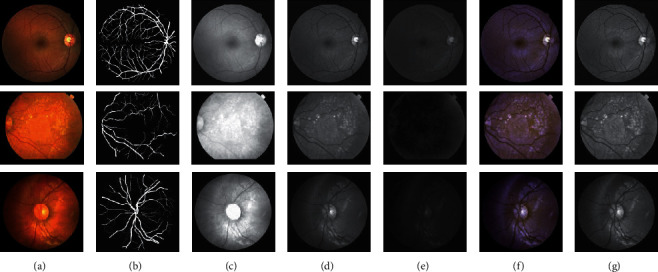
Preprocessing effect diagram. The first row is the DRIVE dataset, the second row is the STARE dataset, and the third row is the CHASE dataset. (a) The original image of each dataset, (b) the corresponding real ground truth, (c) the red (R) channel image of the original image, (d) the green (G) channel image of the original image, (e) the original image for the blue (B) channel image, (f) a preprocessed three-channel image, and (g) a process for converting the preprocessed three-channel image into a grayscale image.

As a means of image processing, ACE technology can effectively improve the visual effect of the image, enhance the recognition rate of information, and highlight differences or characteristics of the target object to extract the target object from the background information more accurately. In deep learning, preprocessing usually makes the input more suitable for a specific application, narrows the range of pixel value intensities, and highlights interesting areas. The reduction of the pixel value intensity range will reduce the amount of training calculations. This paper proposes an image preprocessing method based on ACE technology. Compared with the red or blue channel image in the retinal fundus image, the green channel image shows the best contrast between the retinal vessels and the retinal background. The preprocessing has five steps: (1) extract the green channel in the original image as the first channel; (2) apply contrast-limited adaptive histogram equalization (CLAHE) [26] to the green channel as the second channel; (3) after removing the gamma correction operation on the green channel, it is used as the third channel; (4) combine these three channels to reconstruct a three-channel image; and (5) single-channel grayscale images show better blood vessel background contrast than RGB images [27]. Therefore, the combined three-channel image is converted into a grayscale image.  Figure 2 shows the effect of this preprocessing method.

### 2.3. BFCN

**Figure 3:**
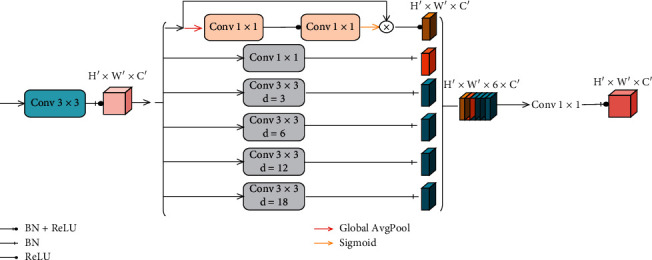
MSIE module structure.

**Figure 4:**
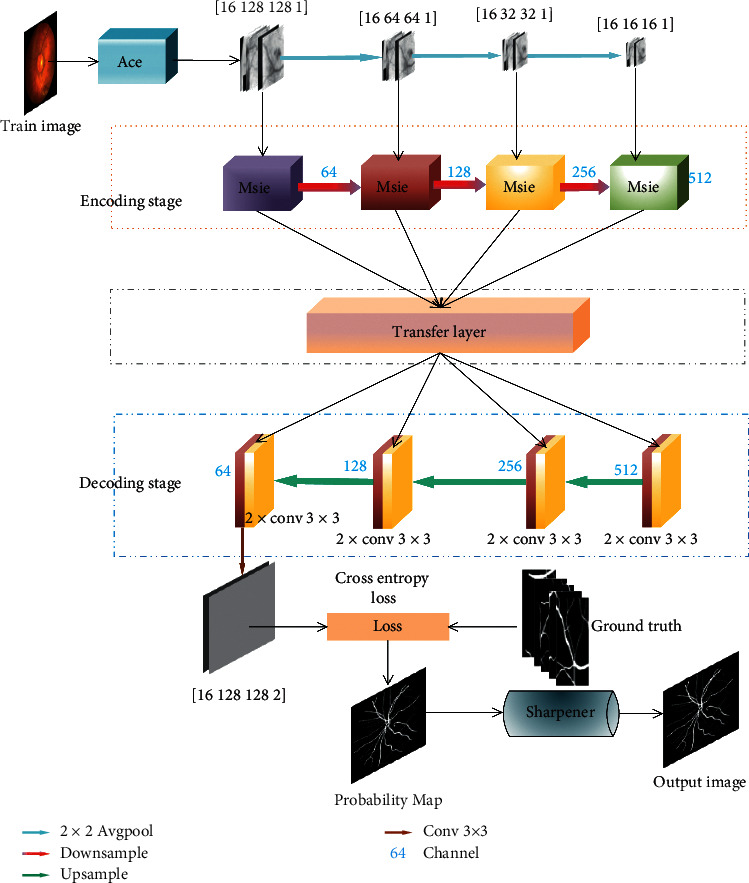
BFCN network structure.

Retinal vascular segmentation is mainly the tension between semantics and location. While global information eases semantic problems, local information can alleviate location problems, and the combination of fine layers and coarse filter layers enables the model to make local predictions without violating global results and minimizes the tension between semantics and location. The BFCN proposed in this paper has a similar overall structure to that of the standard FCN [28], including the encoding process and decoding process, which are symmetrically up and down. The encoding path is capable of encoding low-dimensional input images using richer filters to capture semantic or context information. The decoding path performs upsampling and fusion of low-dimensional features to realize the inverse operation of coding and the restoration of spatial information, so as to achieve precise positioning. The differences between the BFCN and standard FCN are as follows: (1) add side input, which is used to build the input of the image pyramid to realize the fusion of the hierarchical perception field; (2) the encoding process uses a multiscale information extraction (MSIE) module (Figure 3) and multiple convolution layers with different expansion rates instead of a convolution layer to expand the receptive field range without increasing the amount of calculation; and (3) the use of the T_Layer can provide the necessary details and combine the features of the lower layer with that of the higher layer to accurately reconstruct the shape of the segmentation boundary. The network structure is shown in Figure 4.

#### 2.3.1. Receptive Field and Dilated Convolution

**Figure 5:**
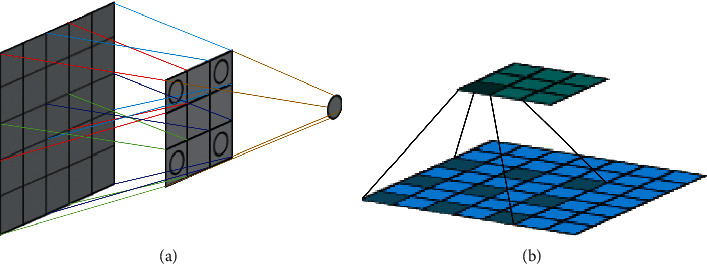
The changing process of the receptive field, (a) the changing process of the ordinary convolution receptive field with a two-layer convolution kernel of 3 × 3, stride of 1, and padding of 0, and (b) the changing process of the dilated convolution receptive field with the convolution kernel of 3 × 3, stride of 1, padding of 0, and expansion rate of 2.

The receptive field is the size of the area on the input image where the pixels on the feature map output by the CNN are mapped. The larger the size of the receptive field is, the larger the size of the receiving field, and the larger the original image range that can be accessed, which also means it may contain more global features with higher semantic hierarchy. On the contrary, the smaller the size is, the more local and detailed the features it contains tend to be. Therefore, the size of the receptive field can be used to roughly judge the abstraction level of each layer, as shown in Figure 5(a).

The traditional FCN is to convolute the image before pooling, reducing the size of the image, and increasing the size of the receptive field. Reducing the image size continuously will cause the loss of information. The advantage of dilated convolution [29] is that under the situation of no loss of information caused by pooling operation, the receptive field size can be increased, and multiscale context information can be captured to make each convolution output contain a larger range of information. The formula for calculating the actual convolution kernel size of dilated convolution (1) is

(1) $$\begin{array}{c} K=k+\left(k-1\right)\times \left(r-1\right). \end{array}$$

In the formula, *k* is the size of the standard convolution kernel, and *r* is the parameter expansion rate of the dilated convolution. When the dilation rate equals to 1, the dilation convolution is the same as the standard convolution. The changing process of the dilated convolution receptive field is shown in Figure 5(b) [30]. Our Figure 4(b) is mainly borrowed from the article [31]. The receptive field of a dilated convolution with a convolution kernel of 3 × 3 and an expansion rate of 2 is equivalent to a normal convolution with a convolution kernel of 5 × 5, without increasing the number of convolution kernel parameters while maintaining the same feature resolution.

#### 2.3.2. MSIE Module

Because the blood vessels in retinal images have different sizes and low contrast with the background, in order to better segment retinal blood vessels of different sizes, the encoding path in this paper uses a richer filter to encode low-dimensional input images to capture semantic and context information. In this paper, the MSIE module uses dilated convolution with different expansion rates for multiscale feature capture to segment blood vessel edges and tiny blood vessels accurately [32]. The MSIE module (Figure 4) contains 4 parallel dilated convolutions with different expansion rates, a common 1 × 1 convolution layer and a feature reweighting layer. Four dilated convolutions reduce cost of computation and the number of parameters while maintaining the good performance. Multiscale context feature information can be captured through dilated convolution with four different expansion rates, while the 1 × 1 convolutional layer retains feature information of the current scale. The feature recalibration layer aims at explicitly establishing the interdependence between the features and the channels, automatically knowing the importance of each channel through learning, and obtaining the global context information of image. The feature reweighting layer first pool the global average of the feature map *X* to transfer each two-dimensional feature channel into a real number *rϵR*. This real number has a global receptive field to some extent, and the number of output channels and the number of channels in the original feature map are the same. Next, change the identification of *R* into *R*′ by the two layers of 1 × 1 convolution, then *R*′′ is output by *R*′ through the sigmoid activation function, and finally *X* multiplied by *R*′′ is output:

(2) $$\begin{array}{c} R\;=\;G\left(X\right)=\overset{C^{\prime }}{\underset{c=0}{\cup }}\left(\sum_{w=0}^{W}\sum_{h=0}^{H}\frac{x_{w,h,c}}{\left(W\times H\right)}\right),\;x\in X\;,\;X\in {ℜ}^{W^{\prime }\times H^{\prime }\times C^{\prime }}, \end{array}$$

(3) $$\begin{array}{c} R^{\prime }=F\left(R\right), \end{array}$$

(4) $$\begin{array}{c} R^{\prime \prime }=S\left(R^{\prime }\right)=\overset{C^{\prime }}{\underset{c=0}{⋃}}\frac{1}{1+e^{-r_{1,1,c}^{\prime }}},\;r^{\prime }\in \prime ,R^{\prime }\in {ℜ}^{1\times 1\times C^{\prime }}, \end{array}$$

(5) $$\begin{array}{c} U=H\left(X,R^{\prime \prime }\right)=\overset{C^{\prime }}{\underset{c=0}{⋃}}\left(\overset{W^{\prime }}{\underset{w=0}{\cup }}\overset{H^{\prime }}{\underset{h=0}{\cup }}x_{w,h,c}\times r_{1,1,c}^{\prime \prime }\right),\;x\in X,\;r^{\prime \prime }\in R^{\prime \prime },\;R^{\prime \prime }\in {ℜ}^{1\times 1\times C^{\prime }},\;U\in {ℜ}^{W^{\prime }\times H^{\prime }\times C^{\prime }}, \end{array}$$

where *X* represents the input feature map, *G*(·) is the global average pooling operation, *F*(·) is the two-layer convolution operation, *S*(·) is the sigmoid activation function, and *H*(·) is the dot product operation. The *U* indicates element-wise value.

#### 2.3.3. Transfer Layer Module

**Figure 6:**
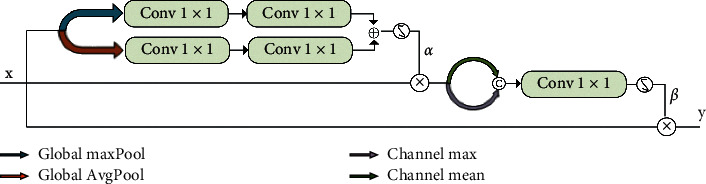
Structure of the transfer layer.

The skip connection in U-Net has the advantages of alleviating the problem of gradient vanishing and repairing the information loss during downsampling [33]. In this paper, the skip connection is added to the transfer layer in the BFCN model, and certain changes are made (Figure 6). Similar to U-Net, except that the transfer layer is embedded in the skip connection, and the output of the coding layer is input to the corresponding decoding layer through the transfer layer. Use the transport layer to improve the sensitivity of the network to information, and at the same time, the effective information of the feature is selected in the encoder to obtain more detailed target information that needs attention and suppress useless information. It is very important for the decoder that has no regulatory information. The proposed transfer layer is shown in Figure 6. It consists of five 1 × 1 convolutional layers, two sigmoid activation functions, two pooling layers, and two channel processing layers. First, perform max-pooling and mean-pooling operations on feature maps, respectively, to output *X*′ and *X*″, and then perform 1 × 1 convolution before their adding results are activated by sigmoid to obtain the gate control coefficient *a*. Next, perform the maximized processing and mean processing of channel on *α* × *X*, respectively, and then perform 1 × 1 convolution before performing sigmoid activation to obtain the gating coefficient *β*. Finally, multiply *X* and *β* to obtain the output *Y* of the transfer layer. Experiments prove that it achieves higher accuracy.

#### 2.3.4. Decoder

The decoder uses deconvolution to upsample the feature map layer by layer, with the upsampling factor of 2, and finally restores to the same resolution as that of the input image. The feature information of the feature map output by MSIE is concatenated with the feature information obtained by deconvolution of the same layer in the decoding path; thereby, the situation where some thin-walled blood vessels and vessel edge information are difficult to recover during upsampling is eliminated. The quality of cascaded feature information is improved by two 3 × 3 convolutional layers. Finally, the eventual segmentation result is output.

#### 2.3.5. Image Postprocessing

Image preprocessing is widely used in deep learning. It is a necessary means to improve model performance. Based on this, the postprocessing of the segmented images is proposed in this paper, aiming at improving the accuracy of retinal blood vessel segmentation. The function of sharpening is to enhance the grayscale contrast, the image edge sharpening process can enhance the grayscale contrast, and the edges and contours in the image are located in the place where the grayscale changes, so the sharpening can enhance the contour edges and details in the image, and a complete object boundary is formed to separate the object from the image. The root cause of the smooth image becoming blurred is that the image has been subjected to averaging or integration operations, so the blurred image can be inversely calculated to make the image clear. In order to make the edges and contour lines that extend in any direction in the middle of the image clearly visible, this article hopes that certain operations on the image are isotropic, and gradient algorithm can meet this requirement, and the gradient algorithm can make the image uniform. The direction of the gradient is the direction of the image change rate. The amplitude ratio of the gradient is equivalent to the difference in grayscale of adjacent pixels. For the image *F* (*x*, *y*), the gradient at the point (*x*, *y*) is defined as

(6) $$\begin{array}{c} \nabla F\left(x,y\right)=\left[\begin{array}{c} \partial F/\partial x\\ \partial F/\partial y \end{array}\right]. \end{array}$$

Its magnitude is

(7) $$\begin{array}{c} \left|\nabla F\left(x,y\right)\right|=\sqrt{{\left(\frac{\partial F}{\partial x}\right)}^{2}+{\left(\frac{\partial F}{\partial y}\right)}^{2}}. \end{array}$$

For discrete images, the differential method of adjacent phase difference is substituted for differentiation; so, formula (6) can be defined as

(8) $$\begin{array}{c} \nabla F\left(x,y\right)\approx \left|\left[F\left(x,y\right)-F\left(x+1,y\right)\right]\right|+{\left|\left[F\left(x,y\right)-F\left(x,y+1\right)\right]\right|}^{2}. \end{array}$$

With the gradient *F*(*x*, *y*), the sharpening result can be obtained according to the gradient. This paper uses the Laplacian algorithm [34]. The Laplacian algorithm is a linear quadratic differential operator. Like the gradient operator, it has rotational invariance. Thereby, the edge sharpening requirements of images in different directions can be met. Including more detailed information, the obtained borders are thinner. Laplacian operator can be defined as

(9) $$\begin{array}{c} \nabla^{2}F\left(x,y\right)=\frac{\partial^{2}F\left(x,y\right)}{\partial x^{2}}+\frac{\partial^{2}F\left(x,y\right)}{\partial y^{2}}. \end{array}$$

Its discrete form is

(10) $$\begin{array}{c} \nabla^{2}F\left(x,y\right)=\;F\left(x+1,y\right)+F\left(x-1,y\right)+F\left(x,y+1\right)+F\left(x,y-1\right)-4F\left(x,y\right). \end{array}$$

Laplacian operator is used to perform sharpening, and the sharpening output *G*(*x*, *y*) is

(11) $$\begin{array}{c} G\left(x,y\right)=F\left(x,y\right)-\nabla^{2}F\left(x,y\right). \end{array}$$

Transform formula (10) into a coefficient form, Laplacian operator,

(12) $$\begin{array}{c} L=\left[\begin{array}{ccc} 0 & -1 & 0\\ -1 & 4 & -1\\ 0 & -1 & 0 \end{array}\right]\overset{\text{Extension template}}{⟶}\left[\begin{array}{ccc} 1 & 1 & 1\\ 1 & -8 & 1\\ 1 & 1 & 1 \end{array}\right]\overset{abs\left(\right)/10}{⟶}\left[\begin{array}{ccc} 0.1 & 0.1 & 0.1\\ 0.1 & 0.8 & 0.1\\ 0.1 & 0.1 & 0.1 \end{array}\right]. \end{array}$$

This paper will sharpen the predicted probability map. The value of each pixel (*x*, *y*) is related to its neighborhood pixel value. The pixel value of (*x*, *y*) is reset by the Laplacian operator that is transformed by the formula (12). The reset pixel value shows a strong relationship with its neighborhood pixel values. It has a certain enhancement effect on the continuity of the end of small blood vessels.

## 3. Experiments

### 3.1. Training and Test Patches

During the training process, 10480 image patches with a size of 128 × 128 were randomly extracted from the training set of each dataset. At the same time, 10480 real label patches with a size of 128 × 128 are extracted from the corresponding real labels at the same location to calculate the loss and train the network. There are 16 images input into the network each time. In testing stage, the image patches are extracted from each tested picture of each dataset, in the sequence of the way of sliding window. The size of the sliding window was 128 × 128, and the sliding stride is 5 pixels. The part of sliding window that exceeds the picture was filled with 0. Similarly, there were 16 images input to the network each time.

### 3.2. Implementation Details

The method in this paper is based on the deep learning open source framework PyTorch [35] and is implemented on a server of an operating system configured with Intel (*R*) Xeon (*R*) E5-2620 V3 2.40 GHz CPU, Tesla K80 GPU, and Ubuntu64. In the training stage, the Adam optimizer [36] (the parameters were set as: *β*_1_ = 0.9, *β*_2_ = 0.999 and *ε* = 10^−8^, and the learning rate *lr* was initialized as 0.001) was used to make the learning rate attenuated by the Plateau [37] method. The training circle was 200, the training batch was 16, and the loss function uses a cross-entropy loss function. It is defined as follows:

(13) $$\begin{array}{c} {\text{Loss}}_{\text{ce}}\left(y,\hat{y}\right)=-\sum y_{i}\log\;{\hat{y}}_{i}+\left(1-y_{i}\right)\log\left(1-{\hat{y}}_{i}\right), \end{array}$$

where *y*_*i*_ represents the real label, and ${\hat{y}}_{i}$ represents the predicted image. Thresholds were set as 0.6, 0.43, and 0.65 when the performance of the DRIVE, STARE, and CHASE datasets is evaluated.

### 3.3. Evaluation Metrics

In order to evaluate the effectiveness of this method for retinal vascular segmentation, the analysis on the performance of sensitivity, specificity, accuracy, and *F*-measure evaluation indicators is performed by making confusion matrix:

(14) $$\begin{array}{c} \text{accuracy}=\frac{\text{TP}+\text{TN}}{\text{TP}+\text{FN}+\text{TN}+\text{FP}}, \end{array}$$

(15) $$\begin{array}{c} \text{sensitivity}=\frac{\text{TP}}{\text{TP}+\text{FN}}, \end{array}$$

(16) $$\begin{array}{c} \text{specificity}=\frac{\text{TN}}{\text{TN}+\text{FP}}, \end{array}$$

(17) $$\begin{array}{c} \text{precision}=\frac{\text{TP}}{\text{TP}+\text{FP}}, \end{array}$$

(18) $$\begin{array}{c} F=2\times \frac{\text{Prec}.\text{Sens}.}{\text{Prec}.+\text{Sens}.}, \end{array}$$

here, TP is the correctly identified blood vessel pixel, and TN is the correctly identified background pixel. FP is the background pixel that is incorrectly segmented into blood vessel pixels, and FN is a blood vessel that is incorrectly marked as a background pixel.

### 3.4. Comparison of Model Improvement Results

The data preprocessing techniques, basic network, and postprocessing of segmented images are combined to verify their effectiveness through using the DRIVE, STARE, and CHASE datasets. In the table, ACE represents an image preprocessing technology of the color enhancement, MSIE is a multiscale information extraction module, T_Layer indicates a conversion layer module, and Sharp indicates a sharpening method of postprocessing the segmented image. The experimental results are shown in Tables 1–3.

Tables 1–3 is the combined verification results of each part on the DRIVE, SATRE, and CHASE datasets, respectively. In Tables 1–3, the experimental results in the first row show that the MSIE module proposed in this paper can effectively segment the retinal blood vessels and achieve good effects. Through the comparison between the first row and the second row, the experimental results show that T_Layer can select the effective feature information in the encoder to obtain more detailed information of the target that needs attention. And with the comparison between the second row and the third row, the experimental results show that the ACE data preprocessing proposed in this paper can have a positive impact on the segmentation of blood vessels, which can make an improvement in accuracy of these three standard datasets. With the comparison between the second row and the forth row, the experimental results show that the sharpening postprocessing of the segmented image proposed in this paper can further process the segmented image to alleviate the fracture problem of small blood vessels in the segmented image and improve the accuracy. The segmentation accuracy is increased by 0.21%, 0.39%, and 0.19% in DRIVE, STARE, and CHASE datasets, respectively. With the comparison between the fifth row and other rows, it shows that the network architecture proposed in this paper can segment the retinal fundus vessels well. Experiment data show that the efficiency of segmentation is the highest after combining all the modules. And the accuracy and *F*-measure on the DRIVE, STARE, and CHASE datasets reached 0.9627/0.8294, 0.9735/0.8442, and 0.9688/0.8102, respectively.

**Figure 7:**
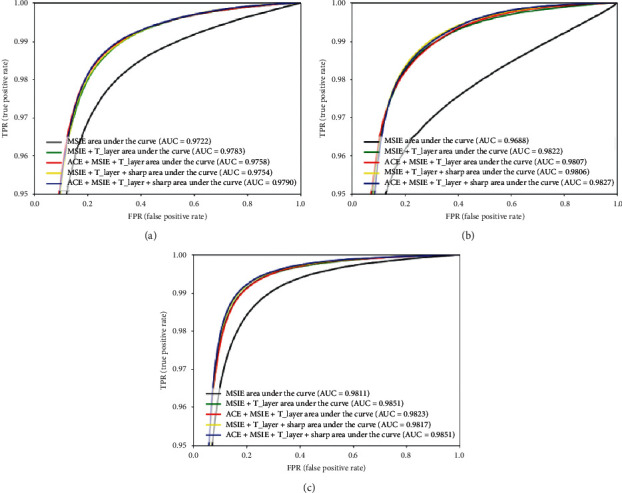
Comparison among ROC curves of the MSIE, T_Layer, ACE, and Sharp modules on the DRIVE, STARE, and CHASE datasets. (a) DRIVE, (b) STARE, and (c) CHASE.

Figure 7 shows the ROC_AUC_ curve analysis after combining the various parts on the DRIVE, STARE, and CHASE datasets. AUC represents the area under the ROC curve. The larger the AUC value is, the more likely the current classification algorithm will rank positive samples before negative samples, to better classify. In Figure 7, the ACE + MSIE + T_Layer + sharp combination has higher ROC_AUC_ value than other combinations. The ROC_AUC_ value on the DRIVE, STARE, and CHASE datasets is 0.9790, 0.9827, and 0.9851, respectively.

**Figure 8:**
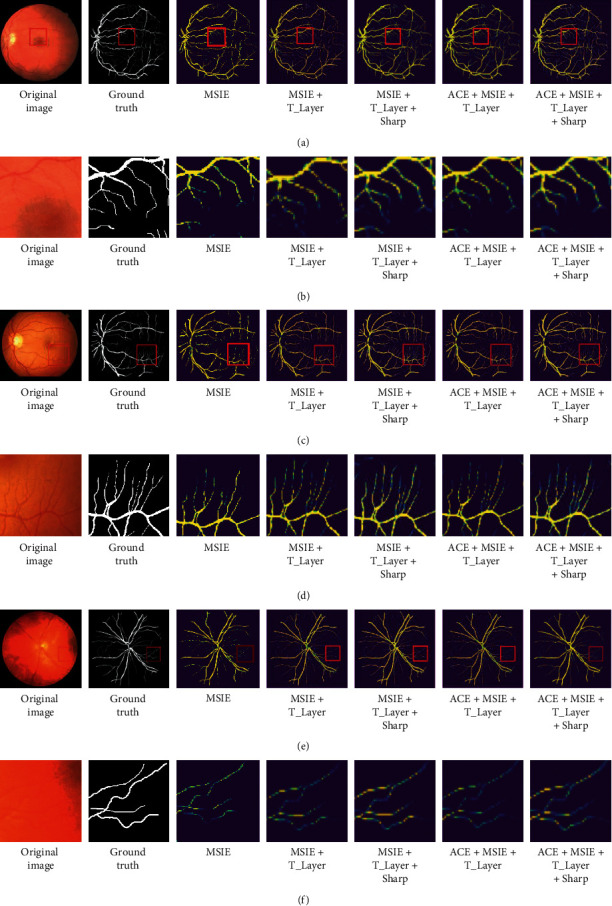
Comparison between model changes and segmentation images. Row (a) is the images of the DRIVE dataset, and row (b) is a local enlargement of the images in row a; row (c) is the images of the STARE dataset, and row (d) is a local enlargement of the images in row (c); row (e) is the images of the CHASE dataset, and row (f) is a local enlargement of the images in row (e). The dark blue in Figure 8 is the background. The yellow in the figure is blood vessels, and the light blue that appears in yellow blood vessels is also blood vessels, but the pixel value of this part of the blood vessel is low, which is easier to segment and break.

In order to further prove the advantages of the data preprocessing technology, basic network, and postprocessing of segmented images proposed in this paper, this paper compares the experimental segmented images combined by each part. In Figure 8, through locally enlarging the corresponding combined segmented images and comparing the locally enlarged images of different combinations, it is obvious that the ACE data preprocessing technology and basic network proposed in this paper are effective for retinal vessel segmentation. Compared with the combination without segmentation image sharpening and postprocessing operation, the combination with these operations has a certain repair effect on the fracture of small blood vessels.

### 3.5. Comparison among Results of Different Segmentation Algorithms

In order to further prove the effectiveness of this method for retinal vessel segmentation, this paper compared the STARE, DRIVE, and CHASE datasets with the methods in some references, respectively, and judged the performance of the vascular segmentation by sensitivity, specificity, accuracy, and *F*-measure.  Table 4 compares the performance of different methods for retinal vessel segmentation on the DRIVE dataset. Compared with references [21, 40, 42–44], the sensitivity index is low, since many background pixels are still classified as vascular pixels by this method in this paper, but the specificity, accuracy, and *F*-measure indicators are all optimal. Compared with the method in the study by Samuel and Veeramalai [21], the specificity of this paper's method was improved by 0.84%.

On the STARE dataset, specificity of the BFCN method has increased by 1.58% compared with that of the method in the study by Samuel and Veeramalai [21] (Table 5). Although the method in this paper does not achieve its highest value in the aspect of *F*-measure and sensitivity, the accuracy rate of the BFCN method is 0.9735, which is 1.26% higher than that of the method in the study by Samuel and Veeramalai [21].

Table 6 compares the performance of different methods for retinal vessel segmentation on the CHASE dataset. Compared with other studies [27, 41–45], the method in this paper reaches the highest value of sensitivity, specificity, accuracy, and *F*-measure. Through the analysis of Tables 4–6, some indicators of the method in this paper have been improved compared with that of the listed references. And especially on the CHASE dataset, the BFCN has reached the highest values of all indicators, which also verifies the effectiveness of the method for retinal vascular segmentation in this paper.

**Figure 9:**
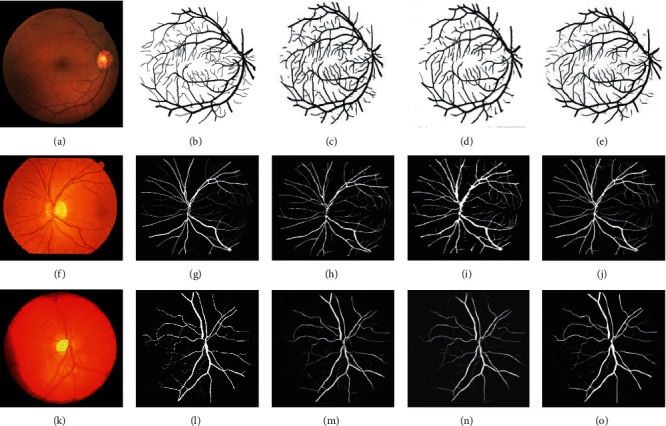
Comparison between the segmented image of the BFCN and other methods on the DRIVE, STARE, and CHASE datasets. (a) Original image, (b) ground truth, (c) method in the study by Yan et al. [41], (d) method in the study by Samuel and Veeramalai [21], (e) ours, (f) original image, (g) ground truth, (h) method in the study by Yan et al. [41], (i) method in the study by Samuel and Veeramalai [21], (j) ours, (k) original image, (l) ground truth, (m) method in the study by Zhuang [43], and (n) method in the study by Wang et al. [44], and (o) ours.

Figure 9 is a comparison among the images of segmentation results of the method in this paper and some references. The first row of images is from the DRIVE dataset, and the second row of images is from the STARE dataset. Both have compared the segmentation results of the BFCN method and that of the methods in the studies by Yan et al. [41] and Samuel and Veeramalai [21]. In the studies by Yan et al. [41] and Samuel and Veeramalai [21], the width of blood vessel extracted by algorithm is so small that many small blood vessels are not reflected and the completion degree of the blood vessel extraction is not high. In medical diagnosis, small blood vessels are of great significance to the retinal image. The loss of small blood vessels will cause much adversity in diagnosis. There is a more complete extraction of retinal blood vessels and a fuller extraction of small blood vessels in this method. The third row of images is from the CHASE dataset. The segmentation results of the BFCN method are compared with the images of the segmentation results of the methods in the studies by Zhuang [43] and Wang et al. [44]. The method in the studies by Zhuang [43] and Wang et al. [44] will generate lots of artifacts, which will cause serious interference to the clinical diagnosis, while the method in this paper generates fewer artifacts. In summary, the method in this paper can effectively and accurately segment retinal blood vessel images.

## 4. Conclusions

This paper proposes a novel end-to-end DCNN architecture called the BFCN for automatic segmentation of retinal blood vessels. In network architecture, ACE data preprocessing technology enhances the contrast between blood vessels and background to improve network performance. The MSIE module uses dilated convolution with different expansion rates and feature recalibration layers to capture the information of retinal blood vessel with different sizes and global context information and reduces the number of parameters to improve model speed. The conversion layer combines shallow information and deep information to recover the lost shallow information and obtain spatial information at the same time. Segmented image postprocessing technology is to further process the segmented probabilistic image to achieve the purpose of repairing the rupture of small blood vessel. Finally, the proposed method is verified by the DRIVE, STARE, and CHASE datasets. Experimental results show that the algorithm proposed in this paper has better performance in retinal vessel segmentation than the multilevel/multiscale DNN method.

## Figures and Tables

**Table 1:** Comparison among the results of DRIVE dataset model changes.

	ACE	MSIE	T_Layer	Sharp	Sensitivity	Specificity	Accuracy	*F*-measure
1		√			0.7453	**0.9845**	0.9540	0.8051
2		√	√		0.7681	0.9820	0.9550	0.8129
3	√	√	√		0.7749	0.9725	0.9586	0.8164
4		√	√	√	**0.8252**	0.9732	0.9571	0.8217
5	√	√	√	√	0.8124	0.9822	**0.9627**	**0.8294**

**Table 2:** Comparison among the results of STARE dataset model changes.

	ACE	MSIE	T_Layer	Sharp	Sensitivity	Specificity	Accuracy	*F*-measure
1		√			0.7163	**0.9911**	0.9625	0.7992
2		√	√		0.7806	0.9806	0.9659	0.8234
3	√	√	√		0.7722	0.9807	0.9666	0.8235
4		√	√	√	0.8274	0.9882	0.9698	0.8334
5	√	√	√	√	**0.8288**	0.9896	**0.9735**	**0.8442**

**Table 3:** Comparison among the results of CHASE dataset model changes.

	ACE	MSIE	T_Layer	Sharp	Sensitivity	Specificity	Accuracy	*F*-measure
1		√			0.7626	0.9845	0.9640	0.7941
2		√	√		0.7900	0.9762	0.9645	0.8070
3	√	√	√		0.7744	0.9782	0.9663	0.8036
4		√	√	√	**0.8440**	0.9836	0.9664	0.8091
5	√	√	√	√	0.8323	**0.9851**	**0.9688**	**0.8102**

**Table 4:** Comparison between the results of the BFCN and other methods on the DRIVE dataset.

Method	Year	Sensitivity	Specificity	Accuracy	*F*-measure
Singh and Srivastava [8]	2016	0.7594	0.9723	0.947	—
Zhang et al. [10]	2015	0.7812	0.9668	0.9504	—
Lázár and Hajdu [38]	2015	0.7646	0.9723	0.9458	—
Zhao et al. [39]	2017	0.782	0.979	0.957	—
Zhang et al. [40]	2017	0.7861	0.9712	0.9527	0.7953
Yan et al. [41]	2018	0.8282	0.9738	0.9609	—
Alom [42]	2018	0.7792	0.9813	0.9556	0.8171
Zhuang [43]	2018	0.7856	0.9810	0.9560	0.8202
Wang et al. [44]	2019	0.7940	0.9816	0.9567	0.8270
Samuel and Veeramalai [21]	2019	0.8282	0.9738	0.9609	—
Ours	2019	0.8124	**0.9822**	**0.9627**	**0.8294**

**Table 5:** Comparison between the results of the BFCN and other methods on the STARE dataset.

Method	Year	Sensitivity	Specificity	Accuracy	*F*-measure
Singh and Srivastava [8]	2016	0.77939	0.9376	0.9270	—
Lázár and Hajdu [38]	2015	0.7248	0.9751	0.9492	—
Zhao et al. [39]	2017	0.789	0.978	0.956	—
Zhang et al. [40]	2017	0.7882	0.9729	0.9547	0.7815
Yan et al. [41]	2018	**0.8979**	0.9701	0.9646	—
Lu [45]	2018	0.8090	0.9770	0.9628	—
Jin et al. [27]	2019	0.7595	0.9878	0.9641	0.8143
Li et al. [46]	2019	0.8465	-	0.9673	0.8435
Samuel and Veeramalai [21]	2019	0.8282	0.9738	0.9609	—
Ours	2019	0.8287	**0.9896**	**0.9735**	0.8442

**Table 6:** Comparison between the results of the BFCN and other methods on the CHASE dataset.

Method	Year	Sensitivity	Specificity	Accuracy	*F*-measure
Zhang et al. [40]	2017	0.7644	0.9716	0.9502	0.7581
Yan et al. [41]	2018	0.7641	0.9806	0.9607	—
Alom [42]	2018	0.7756	0.9802	0.9634	0.7928
Zhuang [43]	2018	0.7978	0.9818	0.9656	0.8031
Wang et al. [44]	2019	0.8074	0.9821	0.9661	0.8037
Lu [45]	2018	0.7571	0.9823	0.9664	—
Jin et al. [27]	2019	0.8155	0.9752	0.9610	0.7883
Ours	2019	**0.8323**	**0.9851**	**0.9688**	**0.8102**

## Data Availability

The data used to support the findings of this study are included within the article.

## References

[1] Soomro T. A., Khan T., Khan M.A.U., Gao J., Paul L., Zheng T. (2018). Impact of ICA-based image enhancement technique on retinal blood vessels segmentation. *IEEE Access*.

[2] Khanal A., Estrada R. (2019). Dynamic deep networks for retinal vessel segmentation. https://arxiv.org/abs/1903.07803.

[3] Dharmawan D. A., Li D., Ng B. P., Rahardja S. (2019). A new hybrid algorithm for retinal vessels segmentation on fundus images. *IEEE Access*.

[4] Yang Y., Huang S., Rao N. (2008). An automatic hybrid method for retinal blood vessel extraction. *International Journal of Applied Mathematics and Computer Science*.

[5] Hoover A. D., Kouznetsova V., Goldbaum M. (2000). Locating blood vessels in retinal images by piecewise threshold probing of a matched filter response. *IEEE Transactions on Medical Imaging*.

[6] Oliveira W. S. (2016). Unsupervised retinal vessel segmentation using combined filters. *PLoS One*.

[7] Dharmawan D., Ng B., Rahardja S. (2018). A modified Dolph-Chebyshev type II function matched filter for retinal vessels segmentation. *Symmetry*.

[8] Singh N. P., Srivastava R. (2016). Retinal blood vessels segmentation by using Gumbel probability distribution function based matched filter. *Computer Methods and Programs in Biomedicine*.

[9] Saffffarzadeh V. M., Osareh A., Shadgar B. (2014). Vessel segmentation in retinal images using multi-scale line operator and K-means clustering. *Journal of Medical Signals and Sensors*.

[10] Zhang L., Fisher M., Wang W. (2017). Retinal vessel segmentation using multi-scale textons derived from keypoints. *Computerized Medical Imaging and Graph*.

[11] Roychowdhury S., Koozekanani D. D., Parhi K. K. (2015). Iterative vessel segmentation of fundus images. *IEEE Transactions on Biomedical Engineering*.

[12] Ali A., Wan Zaki W. M. D., Hussain A. (2019). Retinal blood vessel segmentation from retinal image using B-Cosfire and adaptive thresholding. *Indonesian Journal of Electrical Engineering and Computer Science*.

[13] Chen G. (2017). Retina image vessel segmentation using a hybrid CGLI level set method. *BioMed Research International*.

[14] Wang Y.-B. A novel vessel segmentation in fundus images based on SVM.

[15] Anitha J. Performance improved GA based statistical computing technique for retinal image segmentation.

[16] Ronneberger O., Fischer P., Brox T. U-net: convolutional networks for biomedical image segmentation.

[17] Fu H. Retinal vessel segmentation via deep learning network and fully-connected conditional random fields.

[18] Li Q. (2015). A cross-modality learning approach for vessel segmentation in retinal images. *IEEE Transactions on Medical Imaging*.

[19] Liskowski P., Krawiec K. (2016). Segmenting retinal blood vessels with_newline deep neural networks. *IEEE Transactions on Medical Imaging*.

[20] Lin Y., Zhang H., Hu G. (2018). Automatic retinal vessel segmentation via deeply supervised and smoothly regularized network. *IEEE Access*.

[21] Samuel P. M., Veeramalai T. (2019). Multilevel and multiscale deep neural network for retinal blood vessel segmentation. *Symmetry*.

[22] Fraz M. M., Remagnino P., Hoppe A. (2012). Blood vessel segmentation methodologies in retinal images-a survey. *Computer Methods and Programs in Biomedicine*.

[23] Jiang Y., Tan N., Peng T., Zhang H. (2019). Retinal vessels segmentation based on dilated multi-scale convolutional neural network. *IEEE Access*.

[24] Staal J., Abramoff M. D., Niemeijer M., Viergever M. A., van Ginneken B. (2004). Ridge-based vessel segmentation in color images of the retina. *IEEE Transactions on Medical Imaging*.

[25] Owen C. G., Rudnicka A. R., Mullen R. (2009). Measuring retinal vessel tortuosity in 10-year-old children: validation of the computer-assisted image analysis of the retina (CAIAR) program. *Investigative Opthalmology & Visual Science*.

[26] Reza A. M. (2004). Realization of the contrast limited adaptive histogram equalization (CLAHE) for real-time image enhancement. *The Journal of VLSI Signal Processing-Systems for Signal, Image, and Video Technology*.

[27] Jin Q., Meng Z., Pham T. D., Chen Q., Wei L., Su R. (2019). DUNet: a deformable network for retinal vessel segmentation. *Knowledge-Based Systems*.

[28] Long J., Evan S., Trevor D. Fully convolutional networks for semantic segmentation.

[29] Yu Fisher, Koltun Vladlen, Funkhouser Thomas Dilated residual networks.

[30] Yu F., Koltun V. (2015). Multi-scale context aggregation by dilated convolutions. https://arxiv.org/abs/1511.07122.

[31] Dumoulin V., Visin F. (2016). A guide to convolution arithmetic for deep learning. https://arxiv.org/abs/1603.07285.

[32] Chen Liang-Chieh Encoder-decoder with atrous separable convolution for semantic image segmentation.

[33] Zhou Zongwei Unet++: A nested u-net architecture for medical image segmentation.

[34] Wang Xin (2007). Laplacian operator-based edge detectors. *IEEE transactions on pattern analysis and machine intelligence*.

[35] Paszke A. (2017). Automatic differentiation in pytorch.

[36] Kingma D. P., Jimmy B. A. (2014). Adam: a method for stochastic optimization. https://arxiv.org/abs/1412.6980.

[37] Loshchilov I., Hutter F. (2017). Decoupled weight decay regularization. https://arxiv.org/abs/1711.05101.

[38] Lázár I., Hajdu A. (2015). Segmentation of retinal vessels by means of directional response vector similarity and region growing. *Computers in Biology and Medicine*.

[39] Zhao Y., Zhao J., Yang J. (2017). Saliency driven vasculature segmentation with infinite perimeter active contour model. *Neurocomputing*.

[40] Zhang J., Chen Y., Bekkers E., Wang M., Dashtbozorg B., Romeny B. M. T. H. (2017). Retinal vessel delineation using a brain-inspired wavelet transform and random forest. *Pattern Recognition*.

[41] Yan Z., Yang X., Cheng K.-T. (2018). A three-stage deep learning model for accurate retinal vessel segmentation. *IEEE Journal of Biomedical and Health Informatics*.

[42] Alom M. D. Z. (2018). Recurrent residual convolutional neural network based on u-net (r2u-net) for medical image segmentation. https://arxiv.org/abs/1802.06955.

[43] Zhuang J. (2018). Laddernet: multi-path networks based on u-net for medical image segmentation. https://arxiv.org/abs/1810.07810.

[44] Wang B. O., Qiu S., He H. Dual encoding U-net for retinal vessel segmentation.

[45] Lu J. (2018). A coarse-to-fine fully convolutional neural network for fundus vessel segmentation. *Symmetry*.

[46] Li R., Li M., Li J. (2019). Connection sensitive attention U-NET for accurate retinal vessel segmentation. https://arxiv.org/abs/1903.05558.

