# Corrigendum to “Efficient BFCN for Automatic Retinal Vessel Segmentation”

**DOI:** 10.1155/2020/3942895

**Published:** 2020-11-04

**Authors:** Falin Wang, Yun Jiang, Jing Gao, Wenhuan Liu

**Affiliations:** College of Computer Science and Engineering, Northwest Normal University, Lanzhou, Gansu, China

In the article titled “Efficient BFCN for Automatic Retinal Vessel Segmentation” [1], the order of the authors was incorrect and the corrected authors' list is given above.
